# Human Nonmercaptalbumin Is a New Biomarker of Motor Function

**DOI:** 10.3390/jcm10112464

**Published:** 2021-06-02

**Authors:** Sadayuki Ito, Hiroaki Nakashima, Kei Ando, Kazuyoshi Kobayashi, Masaaki Machino, Taisuke Seki, Shinya Ishizuka, Shunsuke Kanbara, Taro Inoue, Hiroyuki Koshimizu, Ryosuke Fujii, Hiroya Yamada, Yoshitaka Ando, Jun Ueyama, Takaaki Kondo, Koji Suzuki, Yukiharu Hasegawa, Shiro Imagama

**Affiliations:** 1 Department of Orthopaedic Surgery, Graduate School of Medicine, Nagoya University, Nagoya 466-8560, Japan; sadaito@med.nagoya-u.ac.jp (S.I.); andokei@med.nagoya-u.ac.jp (K.A.); k_koba1@f2.dion.ne.jp (K.K.); masaaki_machino_5445_2@yahoo.co.jp (M.M.); taiseki@med.nagoya-u.ac.jp (T.S.); shinyai@med.nagoya-u.ac.jp (S.I.); shunaly0108@gmail.com (S.K.); bluesdrivemonster@hotmail.com (T.I.); love_derika@yahoo.co.jp (H.K.); imagama@med.nagoya-u.ac.jp (S.I.); 2 Department of Preventive Medical Sciences, School of Medical Sciences, Fujita Health University, Toyoake 470-1192, Japan; rfujii@fujita-hu.ac.jp (R.F.); ksuzuki@fujita-hu.ac.jp (K.S.); 3 Department of Hygiene, School of Medicine, Fujita Health University, Toyoake 470-1192, Japan; hyamada@fujita-hu.ac.jp; 4 Department of Biomedical and Analytical Sciences, School of Medical Sciences, Fujita Health University, Toyoake 470-1192, Japan; yando@fujita-hu.ac.jp; 5 Department of Pathophysiological Laboratory Sciences, Graduate School of Medicine, Nagoya University, Nagoya 466-8560, Japan; ueyama@met.nagoya-u.ac.jp (J.U.); taka@met.nagoya-u.ac.jp (T.K.); 6 Department of Rehabilitation, Kansai University of Welfare Science, Osaka 582-0026, Japan; hasegawa@tamateyama.ac.jp

**Keywords:** human nonmercaptalbumin, reduced albumin, oxidative stress, locomotive syndrome

## Abstract

The ratio of human nonmercaptalbumin (HNA) and reduced albumin (HMA) may be a new marker for oxidative stress. Locomotive syndrome (LS) is reduced mobility due to impairment of locomotive organs. We investigated whether the HNA/HMA ratio could be a new biomarker of LS. This study included 306 subjects (mean age 64.24 ± 10.4 years) who underwent LS tests, grip strength, walking speed, and tests for HNA and HMA. Oxidative stress was measured by the ratio of HMA (f(HMA) = (HMA/(HMA + HNA) × 100)), and the subjects were divided into normal (N group; f[HMA] ≥ 70%) and low (L group; f[HMA] < 70%) groups. There were 124 non-elderly (<65 years) and 182 elderly subjects (≥65 years). There were no significant differences in LS, grip strength, and walking speed between the L and N groups in the non-elderly subjects. However, significant differences were found in the elderly subjects. In logistic regression analysis, there was an association between f(HMA) and the LS severity at older ages. LS in the elderly is associated with a decline in HMA and, thus, an increase in oxidative stress. Thus, f(HMA) is a new biomarker of LS.

## 1. Introduction

The majority of developed countries have an aging population [1], and the number of people requiring support and care in their daily lives due to musculoskeletal disorders is increasing [2]. Locomotive syndrome (LS), which is a condition of reduced mobility due to impairment of locomotive organs, was proposed by the Japanese Orthopedic Association (JOA) as an overarching term for this condition [2,3]. LS has received worldwide attention for an assessment of the motor function in motor diseases [4]. LS is associated with a significantly lower quality of life (QOL) [5] and a shorter life expectancy. Prevention of LS has long been advocated for maintaining and improving physical function in middle-aged and elderly people [6].

Oxidative stress reflects the imbalance of reactive oxidative species and antioxidant defenses and plays an important role in the decline of body functions in old age [7,8,9]. Elevated oxidative stress induces apoptosis of skeletal muscle [10], abnormality in neuromuscular junctions [11] and impaired mitochondrial function [12], resulting in decreased muscle performance, one of the major determinants of exercise capacity [13]. A recent systematic review of older adults has shown an association between increased oxidative stress and physical frailty [14]. Given that oxidative stress is one of the origins of age-related decline in functional reserve, the use of biomarkers that reflect the redox status of the body may allow early identification of individuals at risk of functional decline due to musculoskeletal disease. The human serum albumin (HSA) cysteine-34 accounts for about 80% of the extracellular free thiols and is a major extracellular antioxidant [15]. Thus, HSA has been considered an important scavenger of reactive oxidative species, for example hydroxyl radical and hydrogen peroxide [16], but there are reports of differing antioxidative effects of HSA depending on its chemical structure. For example, Cys-34 residue functions as a universal antioxidant residue with excellent scavenging ability against a variety of reactive oxygen species, while Met residue may play an auxiliary role [17,18].

HSAs have been chemically classified into two major categories based on their redox status: human non-mercaptalbumin (HNA: oxidized form) and human mercaptalbumin (HMA: reduced form) [19]. Under oxidative stress, HMA changes to reversibly oxidized HNA-1 and highly oxidized HNA-2. Under oxidative stress, HMA buffers reactive oxidized species and turns them into HNAs; therefore, the proportion of HMA in HSA (f(HMA)) has been considered a biomarker reflecting the redox status of the human body [20]. Although the proportion of each HSA form is generally age- and disease-dependent, studies have shown that HMA, HNA-1, and HNA-2 account for 70–80%, 20–30%, and 2–5%, respectively, of the total albumin in healthy young adults [21].

Several clinical studies have examined the relationship between the redox status of HSA and the severity and progression of hypertension [22,23], obesity [24], liver injury [25], renal function [26,27], anemia [28], and cardiovascular complications in patients on dialysis [29,30]. It is also associated with Diabetes Mellitus [31] and Alzheimer’s disease [32].

Although limited epidemiological studies have analyzed the association between HSA redox status and motor function [33], this redox status might be a biomarker for LS. The purpose of this study was to evaluate the redox status of albumin in a middle-aged and elderly Japanese population, and to investigate its correlation with motor function, including LS.

## 2. Materials and Methods

### 2.1. Study Participants

The individuals surveyed were volunteers who underwent a municipal-supported health checkup in the town of Yakumo in 2016. The town of Yakumo has a population of about 17,000 of whom 28% are over 65 years old. More people are engaged in agriculture and fishing than in urban areas. This town has been conducting annual health checkups since 1982. Physical examinations include voluntary orthopedic and physical function tests, internal examinations, and psychological examinations, as well as a health-related QOL survey (SF-36) [34,35]. This study included all participants who completed an assessment of the LS risk stage. The exclusion criteria were: a history of spine or joint surgery, severe knee injury, severe hip osteoarthritis, history of hip or spine fractures, neuropathy, severe mental illness, diabetes, kidney or heart disease, non-fasting, severe impairment of walking or standing, and impairment of the central or peripheral nervous system.

Of the 555 participants who underwent health checks, 306 (128 men and 178 women) met the inclusion criteria. The research protocol was approved by the Human Research Ethics Committee and the University’s Institutional Review Board (No. 2014-0207). All participants gave written informed consent prior to participation. The research procedure was carried out in accordance with the principles of the Declaration of Helsinki.

### 2.2. Examination of Motor Function

Grip strength in the standing position was measured once for each hand with a hand-grip dynamometer (Toei Light Co., Ltd., Saitama, Japan), and the mean value was used [36]. Subjects walked a straight 10 m course once at their fastest pace, and the time required to complete the course was recorded as the 10 m gait time [37].

### 2.3. LS Stage Tests

To evaluate the risk of LS, the JOA has proposed three tests: the two-step test, the stand-up test, and the 25-question geriatric locomotive function scale (GLFS-25) [2]. LS is categorized into stages 1 and 2, and these tests assess the degree of motor function and define the stages of LS. Stage 1 indicates that movement function has begun to decline, and stage 2 indicates that movement function has progressed towards a decline in mobility.

Three tests were conducted according to the JOA guidelines [2].

In the stand-up test, the ability to stand with a single- or double-leg stance from stools of heights, 40, 30, 20, and 10 cm, is evaluated. The grading of difficulty, from easy to difficult, is in the order of double-leg stance with 40, 30, 20, and 10 cm stools, followed by single-leg stance with 40, 30, 20, and 10 cm. The test result is expressed as the minimum height of the stool that the subject was able to stand up from. 

In the two-step test, a physical therapist measured the length of two steps from the starting line to the tip of the toe. Scores were calculated by normalizing the maximum length of two steps by height.

The GLFS-25 is a self-reported comprehensive survey that refers to the previous month [38]. The scale consists of four questions about pain, 16 questions about Activities of Daily Living (ADL), three questions about social functioning, and two questions about mental status. Each item was graded from no disability (0 points) to severe disability (4 points).

We defined LS0, 1, 2 as follows:

LS0

The subject is categorized as Stage 0 if all three of the conditions are met as follows:

1. Stand-up test, ability to stand on one-leg from a 40-cm-high seat (both legs).
2. Two-step test, >1.3.
3. 25-question GLFS score, <7.

LS1

The subject is categorized as Stage 1 if any of the three conditions are met as follows:

1. Stand-up test, difficulty in one-leg standing from a 40-cm-high seat (either leg).
2. Two-step test, <1.3.
3. 25-question GLFS score, ≥7.

LS2

The subject is categorized as Stage 2 if any of the three conditions are met as follows:

1. Stand-up test, difficulty in standing from a 20-cm-high seat using both legs.
2. Two-step test, <1.1.
3. 25-question GLFS score, ≥16.

### 2.4. Measurements of HSA

During the checkup, fasting blood samples were collected through venipuncture and centrifuged within 1 h of sampling. Serum samples were stored at −80 °C until the assay was performed. Routine biochemical analyses were performed in the laboratory of the Yakumo Town Hospital. Interpersonal measurements of height and weight were taken to calculate the body mass index (BMI, kg/m^2^).

The determination of HSA, HNA, and HMA using high performance liquid chromatography (HPLC) with an ultraviolet detector has been reported by Sogami et al. [39]. In this study, the HPLC-post-column bromocresol green (BCG) method was used, which was engineered to ensure that serum uric acid and bilirubin did not interfere with chromatographic peaks [40]. Frozen serum samples were thawed at room temperature and filtered through a Mini-UniPrep syringe-less filter (Agilent, Tokyo, Japan); HPLC was performed and reacted with BCG reagents to separate HMA and HNA detected at a 620 nm wavelength. The sample volume injected into the HPLC was 5 µL. The mobile phase reagent consisted of *N*-methylpiperazine-HCl buffer (pH 4.5), 40 mM Na_2_SO_4_, and 3% ethanol; the BCG reagent consisted of 150 mM citric acid, 3% Brigi 35, and 0.3 mM BCG. For all experiments, distilled water deionized to 18 mΩ using the Millipore Milli-Q System (Millipore Co., Bedford, MA, USA) was used.

The HPLC system used in this study was the Hitachi Lacrom Ice System (Hitachi, Tokyo, Japan), which consisted of an isocratic pump (L-2130), an auto-injector (L-2200), and a column oven (L-2300). Chromatograms were obtained using a photodiode array detection system (model L-2455). A Shodex Asahipak GS-570 GS column (100 mm × 7.5 mm ID) was used to separate the HSA components before sample injection.

In the present experiment, the peak of HNA-2 was not sufficiently quantified, and its peak area was not considered in subsequent analyses. To numerically assess the redox state of HSA from the HPLC profile, f(HMA), which represents the ratio of the peak area of HMA to the peak area of HSA, has been used in previous similar studies [41]. Hence, we followed these reports for the present study.

The f(HMA) was calculated using the following equation: f(HMA) = HMA area/(HMA area + HNA area) × 100.

Previous studies have demonstrated that f(HMA) accounts for 70–80% of the total albumin in a healthy young adult [42]. Therefore, the cut-off value of f(HMA) was determined to be 70%. We divided the participants into the normal (N, f(HMA) ≥ 70%) and lower (L, f(HMA) < 70%) oxidative stress groups.

### 2.5. Statistical Analysis

Continuous variables were expressed as mean ± standard deviation (SD). We compared continuous variables of the L group to those of the N group using the student t-test, and categorical variables of the L group to those of the N group using the Chi-squared test. Logistic regression analysis was performed to evaluate important risk factors of elevated oxidative stress, as defined by f(HMA) < 70%: L group. The dependent variable was N versus L groups. Following univariable analysis, variables that yielded a *p*-value < 0.20 were included in the multivariable analysis.

Each analysis was done separately for the under-65 (non-elderly) and the over-65 (elderly) groups.

All statistical analyses were performed using SPSS Statistics v.22.0 software for Mac (IBM Corp., Armonk, NY, USA). A *p*-value < 0.05 was considered significant in all analyses.

## 3. Results

Table 1 shows the participant characteristics. The participants had an average age of 64.24 ± 10.4 years; 128 were male and 178 were female. The mean albumin serum level was 4.39 ± 0.25 (g/dL); The mean f(HMA) was 69.49 ± 7.02%; there were 151 and 155 participants in the N and L groups, respectively. With respect to the severity of LS, 118 participants (38.6%) were at no risk (stage 0), 116 (37.9%) were stage 1, and 72 (23.5%) were stage 2 [43].

### 3.1. Non-Elderly Participants

The average age was 54.19 ± 7.34 years, and the mean f(HMA) was 72.96 ± 5.86%. Eighty-four (67.7%) and 40 (32.3%) subjects were considered to be in the N and L oxidative stress groups, respectively. In terms of LS, 52 (41.9%), 50 (40.3%), and 22 participants (17.8%) were grouped into no risk (stage 0), stage 1, and stage 2, respectively (Table 1). The average age was significantly higher in the L group (N: 53.42 ± 7.5, L: 56.89 ± 6.15, *p* < 0.001). Gender, height, weight, BMI, grip strength, and gait speed were not significantly different between the groups. There was no significant difference in the severity of LS between the N and L oxidative stress groups (stage 0: 41.7 and 42.5%, stage 1: 40.5 and 40.0%, and stage 2: 17.8 and 17.5% in the N and L groups, *p* = 0.90) (Table 2).

### 3.2. Elderly Participants

The average age was 71.19 ± 5.24 years, and the mean f(HMA) was 67.09 ± 6.76%. Sixty-seven (36.8%) and 115 (63.2%) subjects were categorized in the N and L oxidative stress groups, respectively. In terms of LS, 66 (36.3%), 66 (36.3%), and 50 (27.4%) participants were grouped into stage 0, stage 1, and stage 2, respectively (Table 1).

Age and BMI were not significantly different between the N and L oxidative stress groups. There were significant differences in the percentage of LS in elderly participants (N: stage 0: 33 (49.3%), stage 1: 21 (31.3%), stage 2: 13 (19.4%); L: stage 0: 33 (28.7%), stage 1: 45 (39.1%), stage 2: 37 (32.2%); *p* = 0.004). There were significant differences in gender, height, weight, grip strength, and gait speed in elderly participants (*p* < 0.001, *p* < 0.001, *p* = 0.018, *p* < 0.001, *p* = 0.002, respectively) (Table 3).

Since there were several factors with significant differences, they were examined as covariates for risk factors for elevated oxidative stress, as defined by f(HMA) > 70%: L group in logistic regression analysis, which found only LS as a risk factor for elevated oxidative stress (OR 0.515, 95% confidence interval, 95% CI: 0.281–0.943, *p* = 0.032) (Table 4). As the LS stage increased by 1, the risk of becoming L increased by 0.515 times.

## 4. Discussion

There have been several reports of the association between f(HMA), a marker of oxidative stress, and chronic diseases [16]. However, few reports have indicated its association with motor function [33]. Furthermore, to our knowledge, this is the first study to report on the association between f(HMA) and LS. The present study indicated that subjects with more severe LS stages had higher oxidative stress as assessed by f(HMA) levels in the elderly group, where oxidative stress was associated with a decline in locomotive function. The f(HMA) ratio could be a new biomarker associated with LS in elderly subjects.

Oxidative stress reflects the imbalance of reactive oxidative species and antioxidant defenses, and it plays an important role in the decline of body functions [7]. It has been reported that oxidative stress is associated with chronic diseases, including hypertension, obesity, liver injury, renal function, anemia, and cardiovascular complications [30]. Furthermore, increased oxidative stress in the elderly might be associated with a deterioration in motor function as increased oxidative stress reduces walking speed in elderly women [33]. This study concurred, showing increased oxidative stress and reduced motor functions, such as grip strength and walking speed, in elderly people, which worsened the degree of impairment of locomotion.

There are several possible mechanisms to explain the association between f(HMA) levels and motor performance in LS. Oxidative stress is associated with muscle function through several pathways, including changes in neuromuscular junctions, reduced muscle energy metabolism, and reduced calcium release from the endoplasmic reticulum [11]. Another possible mechanism is muscle atrophy from increased proteolysis and decreased protein synthesis due to increased oxidative stress [11]. Oxidative stress has been reported to impair skeletal muscle as well as cardiovascular energy metabolism [11]. The association between f(HMA) and exercise capacity may be explained by the effect of oxidative stress on these systemic factors that determine exercise capacity.

In multivariate analysis, f(HMA) was associated with locomotion rather than simple motor functions, such as grip strength and gait, in the elderly group. LS is the concept of functional decline due to problems in bone, cartilage, muscle, and nerves [44], and f(HMA) may be related to abnormalities in these motor organs. However, an association between f(HMA) and decline in motor function or LS was not found in the non-elderly. The simple increase in oxidative stress does not affect motor function, but long-term exposure to oxidative stress, such as with age-related chronic inflammation, may be associated with a functional decline [45].

LS is a condition that requires nursing care. As it is a motor disease that is expected to improve with locomotion training, early detection leads to the preclusion of unnecessary nursing care [44]. In the current study, the oxidative stress marker f(HMA) was found to be associated with the degree of LS, and it may therefore be a new biomarker for the early detection of LS. If this is confirmed, it will be possible to intervene in LS from an early stage, resulting in nursing care being unnecessary. Furthermore, as exercise testing to diagnose LS is difficult in the limited time in an outpatient setting, if f(HMA) becomes a biomarker for suspected LS, it could provide a simple objective diagnostic modality for LS.

The modifiability of f(HMA) by intervention, and its responsiveness to changes in motor performance, need to be investigated in future studies. Supplementation with branched-chain amino acids is a potential intervention to increase f(HMA) levels. A previous study in patients with cirrhosis showed that administration of branched-chain amino acids increased f(HMA) [19]. LS may be improved by nutritional therapies that improve f(HMA), which may be a potential new treatment other than exercise therapy. There is scope to consider the link between LS, f(HMA), and nutritional status.

It may also be possible that the exercise regimens reported so far to improve LS may improve f(HMA) and reduce oxidative stress. Therefore, the results of this study may provide a basis for the hypothesis that exercise therapy, as previously described, improves systemic diseases caused by oxidative stress [46].

This study has several potential limitations. First, the participants were middle-aged and elderly people who lived in a relatively rural area, where many had jobs in agriculture or fishing. Thus, the lifestyle of these subjects differed from that of people in an urban environment. Furthermore, the participants attended for annual health examinations, which suggests that they may be more health conscious than other people. Second, this was a cross-sectional, single-center study. In the future, longitudinal and multicenter collaborative research will be needed to verify our findings. Finally, the specificity of f(HMA) as a biomarker of LS could not be discussed, because other biomarkers related to oxidative stress were not measured. Nevertheless, the present study still has a clinical application, by indicating that the redox state of HSA might serve as a biomarker for LS.

## 5. Conclusions

In conclusion, this study demonstrated that f(HMA), a marker for the redox state of HSA, correlated with the severity of LS in elderly people. Thus, we suggest that f(HMA) could be a novel biomarker of LS.

## Figures and Tables

**Table 1:** Demographics and clinical characteristics.

Characteristics	Total (*n* = 306)	Non-Elderly (*n* = 124)	Elderly (*n* = 182)
male/female	128/178	40/84	88/94
Age (years old)	64.24 ± 10.4	54.19 ± 7.34	71.19 ± 5.24
Height (cm)	157.88 ± 8.15	159.56 ± 8.16	156.71 ± 7.97
Weight (kg)	58.84 ± 11.35	60.04 ± 12.61	58.02 ± 10.36
BMI (kg/cm^2^)	23.5 ± 3.48	23.44 ± 3.73	23.54 ± 3.31
grip strength (kg)	27.06 ± 8.88	27.28 ± 9.47	26.9 ± 8.46
gait speed (m/s)	1.88 ± 0.29	1.94 ± 0.28	1.84 ± 0.29
Albumin (g/dL)	4.39 ± 0.25	4.42 ± 0.25	4.36 ± 0.26
f(HMA) (%)	69.49 ± 7.02	72.96 ± 5.86	67.09 ± 6.76
N/L	151/155	84/40	67/115
Stage of LS (0/1/2)	118/116/72	52/50/22	66/66/50

BMI: body mass index; f(HMA): fraction of human mercaptalbumin; f(HMA) = HMA area/(HMA area + HNA area); HMA: human mercaptalbumin; N: participants with f(HMA) of 70% or more; L: participants with f(HMA) less than 70%; LS: locomotive syndrome. Values are mean ± SD for each group.

**Table 2:** The comparison of each parameter between the N and L groups in non-elderly participants.

Non-Elderly (*n* = 124)	N Group (*n* = 84)	L Group (*n* = 40)	*p*
male/female	32/52	16/24	0.304
Age (years)	53.42 ± 7.5	56.89 ± 6.15	<0.001
Height (cm)	160.76 ± 7.87	159.47 ± 8.45	0.579
Weight (kg)	59.28 ± 11.48	61.8 ± 12.28	0.283
BMI (kg/cm^2^)	22.9 ± 3.52	24.27 ± 3.84	0.69
grip strength (kg)	28.44 ± 9.55	27.57 ± 9.63	0.639
gait speed (m/s)	1.95 ± 0.27	1.92 ± 0.31	0.623
Stage of LS (0/1/2)	35/34/15	17/16/7	0.904

BMI: body mass index; N group: participants with f(HMA) of 70% or more; L group: participants with f(HMA) less than 70%; LS: locomotive syndrome. Values are mean ± SD for each group.

**Table 3:** The comparison of each parameter between the N and L groups in elderly participants.

Elderly (*n* = 182)	N Group (*n* = 67)	L Group (*n* = 115)	*p*
male/female	40/27	47/68	<0.001
Age (years)	69.95 ± 4.41	71.55 ± 5.63	0.057
Height (cm)	159.13 ± 7.94	155.32 ± 7.81	<0.001
Weight (kg)	59.11 ± 10.2	57.37 ± 10.43	0.018
BMI (kg/cm^2^)	23.25 ± 3	23.69 ± 3.42	0.930
grip strength (kg)	28.35 ± 8.51	25.8 ± 8.39	<0.001
gait speed (m/s)	1.92 ± 0.24	1.78 ± 0.3	0.002
Stage of LS (0/1/2)	33/21/13	33/45/37	0.004

BMI: body mass index; N group: participants with f(HMA) of 70% or more; L group: participants with f(HMA) less than 70%; LS: locomotive syndrome. Values are mean ± SD for each group.

**Table 4:** Logistic regression analysis for risk factors of the elevation of oxidative stress (L group) in elderly participants.

Elderly	B	SE	Wald	df	*p*	OR	95% CI
male/female	0.031	0.709	0.002	1	0.966	1.031	0.257–4.136
Age (years)	−0.05	0.042	1.412	1	0.235	0.951	0.876–1.033
Height (cm)	0.079	0.042	3.482	1	0.062	1.082	0.996–1.175
Weight (kg)	−0.005	0.025	0.043	1	0.836	0.995	0.947–1.045
grip strength (kg)	−0.08	0.755	0.011	1	0.915	0.923	0.21–4.054
gait speed (m/s)	−0.003	0.039	0.007	1	0.932	0.997	0.924–1.075
Stage of LS (0/1/2)	−0.663	0.309	4.62	1	0.032	0.515	0.281–0.943

OR: odds ratio; CI: confidence interval; L group: participants with f(HMA) less than 70%; LS: locomotive syndrome.

## Data Availability

The data of health checkups used to support the findings of this study are available from the corresponding author upon request.
